# Early prediction of treatment outcome for lenvatinib using ^18^F-FDG PET/CT in patients with unresectable or advanced thyroid carcinoma refractory to radioiodine treatment: a prospective, multicentre, non-randomised study

**DOI:** 10.1186/s13550-023-01019-9

**Published:** 2023-07-17

**Authors:** Satoshi Takeuchi, Kenji Hirata, Keiichi Magota, Shiro Watanabe, Rika Moku, Akihiko Shiiya, Jun Taguchi, Shin Ariga, Tomohiro Goda, Yoshihito Ohhara, Takurou Noguchi, Yasushi Shimizu, Ichiro Kinoshita, Rio Honma, Yasushi Tsuji, Akihiro Homma, Hirotoshi Dosaka-Akita

**Affiliations:** 1 Department of Medical Oncology, Faculty of Medicine and Graduate School of Medicine, Hokkaido University, Sapporo, Japan; 2 Department of Diagnostic Imaging, Hokkaido University Graduate School of Medicine, Sapporo, Japan; 3 Division of Medical Imaging and Technology, Hokkaido University Hospital, Sapporo, Japan; 4 Department of Nuclear Medicine, Hokkaido University Hospital, Sapporo, Japan; 5 Department of Medical Oncology, Tonan Hospital, Sapporo, Japan; 6 Department of Otolaryngology-Head and Neck Surgery, Hokkaido University Graduate School of Medicine, Sapporo, Japan

**Keywords:** Lenvatinib, Early prediction, Treatment outcome, ^18^F-FDG PET/CT, Differentiated thyroid carcinoma

## Abstract

**Background:**

Lenvatinib is widely used to treat unresectable and advanced thyroid carcinomas. We aimed to determine whether ^18^F-fluorodeoxyglucose (FDG) positron emission tomography/computed tomography (PET/CT) performed 1 week after lenvatinib treatment initiation could predict treatment outcomes.

**Results:**

This was a prospective, nonrandomised, multicentre study. Patients with pathologically confirmed differentiated thyroid carcinoma (DTC) and lesions refractory to radioiodine treatment were eligible for inclusion. Patients were treated with 24 mg lenvatinib as the initial dose and underwent PET/CT examination 1 week after treatment initiation. Contrast-enhanced CT was scheduled at least 4 weeks later as the gold standard for evaluation. The primary endpoint was to evaluate the discrimination power of maximum standardised uptake value (SUVmax) obtained by PET/CT compared to that obtained by contrast-enhanced CT. Evaluation was performed using the area under the receiver operating characteristic (ROC-AUC) curve. Twenty-one patients were included in this analysis. Receiver operating characteristic (ROC) curve analysis yielded an AUC of 0.714 for SUVmax after 1 week of lenvatinib treatment. The best cut-off value for the treatment response for SUVmax was 15.211. The sensitivity and specificity of this cut-off value were 0.583 and 0.857, respectively. The median progression-free survival was 26.3 months in patients with an under-cut-off value and 19.7 months in patients with an over-cut-off value (*P* = 0.078).

**Conclusions:**

The therapeutic effects of lenvatinib were detected earlier than those of CT because of decreased FDG uptake on PET/CT. PET/CT examination 1 week after the initiation of lenvatinib treatment may predict treatment outcomes in patients with DTC.

*Trial registration*: This trial was registered in the University Hospital Medical Information Network (UMIN) Clinical Trials Registry (number UMIN000022592) on 6 June, 2016.

## Background

The incidence of thyroid carcinoma has steadily increased [1]. In 2012, approximately 298,000 individuals were estimated to have been newly diagnosed with thyroid carcinoma worldwide [2]. There are several histological types of thyroid carcinoma: differentiated (including papillary, follicular, and Hürthle cells), medullary, and anaplastic [3]. Among them, differentiated thyroid carcinoma (DTC) accounts for 80–90% of all thyroid carcinomas. In 2013, DTC accounted for a total annual cost of approximately $1.6 billion annually in the United States [4]. Nevertheless the incidence of thyroid cancer has declined since 2014 [5].

Surgical resection of the entire tumour is the primary initial treatment. In addition, it is important to remove the remaining thyroid gland. Thyroid-stimulating hormone (TSH) suppression is a standard therapy after surgery. The recurrence rate in patients with DTC is 7–23% [6]. Radioactive iodine is usually administered immediately after surgery or after detection of metastatic disease. Chemotherapy is administered to patients with DTC that is refractory to radioiodine treatment. Although traditional cytotoxic agents have limited efficacy, advances in molecular genetics have rendered DTC treatable by targeting various oncogenic pathways [7]. Among these, two oral molecular-targeted drugs, sorafenib and lenvatinib, have emerged in recent years [8]. However, molecular-targeted drugs have several drawbacks, such as high cost, unique adverse events, and the lack of a method to predict treatment outcomes. Although no trial has compared these two drugs, lenvatinib has a better response rate (RR) and progression-free survival (PFS) than sorafenib [9, 10]. Thus, lenvatinib is more frequently used in daily practice because of its high efficacy.

Nuclear imaging, including ^18^F-fluorodeoxyglucose (FDG) positron emission tomography/computed tomography (PET/CT), is more suitable than conventional CT for early response evaluation because PET/CT visualises pathophysiological functions, whereas CT evaluation is based on tumour size. FDG reflects glucose metabolism, which is controlled by the phosphatidylinositol-3-kinase (PI3K)/Akt (also known as protein kinase B) pathway. As lenvatinib inhibits the upstream pathway [11], its therapeutic effects can be detected as a decrease in FDG uptake on PET/CT.

The aim of this study was to determine whether PET/CT 1 week after lenvatinib treatment initiation is useful in predicting the treatment outcome of lenvatinib in patients with unresectable or advanced DTC refractory to radioiodine treatment.

## Methods

### Study population

The key eligibility criteria were as follows: age > 18 years, Eastern Cooperative Oncology Group performance status (ECOG PS) 0–2, measurable pathologically confirmed differentiated thyroid carcinoma, lesions refractory to radioiodine treatment, and no prior therapy with tyrosine kinase inhibitors, including sorafenib and lenvatinib. The definition of a disease refractory to radioiodine treatment is described later. The exclusion criteria were as follows: medullary or anaplastic carcinoma, prior treatment with chemotherapy, synchronous malignancies except for early-stage lesions curable by endoscopy, fasting blood glucose > 150 mg/dL, ECOG PS 3–4, thromboembolism requiring treatment, pregnant or nursing women, and other reasons as judged by the investigator.

### Study design

This study was conducted prospectively as a non-randomised multicentre study. All relevant institutional review boards approved the study protocol. All patients provided written informed consent before participation. This trial was registered in the University Hospital Medical Information Network (UMIN) Clinical Trials Registry (number 000022592). This study was conducted in accordance with the principles of the Declaration of Helsinki. We recruited patients from 1 June 2016 to 31 March 2020. Data were collected and analysed by the investigators.

**Fig. 1:**
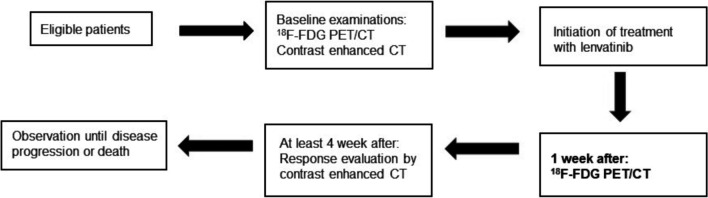
Flowchart of the study. ^18^F-FDG PET/CT: ^18^F-fluorodeoxyglucose positron emission tomography/Computed tomography

Eligible patients underwent FDG PET/CT, contrast-enhanced CT, and blood tests at baseline within 28 days of treatment initiation. Eligible patients were orally administered lenvatinib 24 mg once daily. One week after treatment initiation, the patients underwent FDG PET/CT and blood tests. Contrast-enhanced CT was performed at least 4 weeks after treatment initiation as the gold standard for evaluation. The treatment response was further confirmed by additional CT performed at least 8 weeks later. The patients were observed until disease progression or death occurred. The procedure is summarised in Fig. 1.

### Refractoriness to radioiodine treatment

In this study, the patient was considered refractory to radioiodine treatment if they presented any of the following:

1. At least one measurable lesion without iodine uptake on any radioiodine scan;
2. At least one measurable lesion progressed after radioiodine treatment, despite radioiodine avidity at the time of treatment;
3. Cumulative activity of radioiodine was > 400 mCi (14.8 GBq)

### Primary endpoint

The highest metabolic activity within the tumour, that is, the maximum standardised uptake value (SUVmax) obtained by PET/CT, is considered the most common marker of metabolic changes in the tumour. As the main purpose of this study was to determine whether the change in SUVmax at an early time point after initiation of treatment can predict treatment outcome, the primary endpoint was to evaluate the discrimination power of SUVmax obtained by PET/CT performed 1 week after initiation of lenvatinib treatment compared with the response evaluation obtained by contrast-enhanced CT performed at least 4 weeks after treatment initiation. Evaluation was performed using the area under the receiver operating characteristic (ROC-AUC) curve.

### Secondary endpoint

The secondary endpoint was to determine the optimal SUVmax cut-off value which determined the closest distance from the upper left corner to the ROC curve obtained from the baseline PET/CT. The other endpoint was to perform the analysis using total lesion glycolysis (TLG), the product of mean SUV and metabolic tumour volume (MTV). MTV is the sum of the tumour volumes that show a higher SUV than a certain threshold. In this study, the previously proposed semi-automated method was used to measure the SUV of the liver to determine the threshold value [12]. The threshold was determined using the SUVmean and its standard deviation (SD) in the liver’s VOI as follows: threshold = SUVmean + 3 × SD [13].

### Image acquisition and evaluation

Although this was a multicentre study, all PET/CT scans were performed at the Hokkaido University Hospital using the same PET/CT device. FDG was produced using an in-house cyclotron or was purchased from Nihon Medi-Physics Co. Ltd. (Tokyo, Japan). Patients were instructed to fast for at least 6 h before FDG administration to achieve a blood glucose level < 150 mg/dL. Blood glucose levels were measured prior to FDG administration. ^18^F-FDG (3.7 MBq/kg) was administered intravenously, and patients were allowed to rest in a quiet and dimly lit room. Approximately 50 min after ^18^F-FDG injection, an integrated PET/CT system was used to acquire the imaging data (Gemini TF PET/CT scanner; Philips Healthcare, OH, USA). PET/CT was performed in accordance with guidelines published by the National Cancer Institute [14]. PET/CT scanning is typically performed from the level of the vertex of the skull or orbits through the upper thighs, unless the patient has known metastatic lesions (e.g. tibial lesions). Whole-body unenhanced CT was performed before emission acquisition for anatomical localisation and attenuation correction. The CT parameters included an axial slice thickness of 2 mm, tube voltage of 120 kV, fixed (120 mA) or variable tube current, and table speed of 13.5 mm/s. Emission data were acquired in three-dimensional mode using a state-of-the-art time-of-flight technique and stored in list mode. Emission scanning was initiated 60 min (allowance: 55–65 min) after FDG administration for 2 min per bed position. The trans axial and axial fields of view were 576 and 180 mm, respectively. PET images were reconstructed for attenuation-corrected and non-attenuation-corrected data using a blob-based, ordered-subset, expectation–maximisation algorithm. The PET, CT, and fused images were displayed in 4-mm slices. Contrast-enhanced CT examinations were performed at each institution. The reconstructed image data were stored electrically in the hospital information system at the Hokkaido University Hospital. The list-mode data were electronically stored in the Department of Radiology at Hokkaido University Hospital.

### Image review and tumour analysis

FDG PET/CT and contrast-enhanced CT data were interpreted clinically by a board-certified nuclear medicine physician and a diagnostic radiologist, respectively, at our institution. For FDG PET/CT analysis, a non-physiological increase in FDG uptake compared with the surrounding background was classified as positive for the disease. A nuclear medicine physician measured the SUVmax within the tumour using a spherical volume of interest. The SUV was defined as the measured activity concentration (kBq/mL) multiplied by body weight (g) divided by the injected activity (kBq). Contrast-enhanced CT images were analysed using the Response Evaluation Criteria for Solid Tumours (RECIST) criteria.

### Sample size

As the response rate to lenvatinib was reported to be 64.8% [10], we assumed the expected response rate to lenvatinib in this study to be 60%. The significance level was set at 5%. Using Obchowski’s method [15] with a diagnostic ability of SUVmax area under the curve (AUC) = 0.84, 20 patients were required: 12 responders and 8 non-responders, to achieve a power of 80%. As a 10% loss to follow-up or discontinuation of treatment within 1 week from the initiation of lenvatinib treatment was expected, we included 21 patients.

### Statistical considerations

Statistical analyses were performed using RStudio (version 1.1, RStudio Inc., Boston, MA, USA). ROC curve analysis was performed to assess the discriminatory power of PET/CT 1 week after treatment initiation. The ROC-AUC curve was calculated using the trapezoidal method. Then, the 95% confidence interval (CI) was calculated using the bootstrap method. For the secondary endpoints, the optimal cut-off value was calculated using the Youden Index. A similar analysis was performed for the TLG. PFS and 95% CIs were calculated using Kaplan–Meier curves. Survival curves were obtained using Kaplan–Meier analysis and compared using log-rank tests. Statistical significance was set at *P* < 0.05. Statistical analyses were performed by Satista Co. Ltd. (Kyoto, Japan).

## Results

### Study population

Twenty-two patients were recruited from 1 June 2016 to 31 March 2020. The survival data cut-off date was 30 April 2022. One patient was excluded after registration because tumour invasion into the internal carotid artery was detected during the pre-treatment evaluation. Although the patient was in good general condition, lenvatinib treatment was discontinued because tumour invasion could be a serious risk factor for fistula or bleeding [16]. Therefore, twenty-one patients were included for analysis. Patient characteristics are presented in Table 1. The median length of follow-up time was 32.5 months (range 4.7–70.4 months). The median patient age was 69 years (range 55–82). The patients were in good general condition, with an ECOG PS 0 or 1. All the patients received TSH-suppressive therapy for cancer management. Median TSH at treatment was 0.45 μIU/mL (range < 0.01–15.61, normal range 0.35–4.94). The thyroglobulin test is used as a tumour marker to assess the aggressiveness of thyroid cancer. The median thyroglobulin level at treatment initiation was 378.2 ng/mL (range 1.45–70,909, normal range < 46 ng/mL). All the patients had distant metastases. The most frequent metastases were in the lungs and lymph nodes (15 patients in each group). All patients had papillary carcinoma. The median SUVmax and TLG before treatment were 27.7 and 376.5, respectively.

**Table 1:** Patient characteristics

Characteristics		All patients (n = 21)
Gender	Male	10 (48%)
	Female	11 (52%)
Age	Median (range)	69.6 (55 – 82)
ECOG PS*	0	7 (33%)
	1	13 (62%)
	2	1 (5%)
Metastatic site (duplication)	Lymph node	14 (67%)
	Lung	14 (67%)
	Bone	6 (29%)
	Others**	4 (19%)
Histology	Papillary	21 (100%)
Median TSH (range)		0.45 μIU/mL (< 0.01 – 15.61)
Median FT3 (range)		2.3 pg/dL (1.6 – 11.3)
Median FT4 (range)		1.8 ng/mL (0.9 – 3.5)
Thyroglobrin (range)		378.2 ng/mL (1.5 – 70,909)
Median SUVmax (range)		27.7 (5.3 – 84.8)
Median MTV (range)		48.4 (1.4 – 471.4)
Median TLG (range)		376.5 (5.7 – 2181.8)

^*^Eastern cooperative oncology group performance status

^**^Others included adrenal, pancreas, pleura, and brain

### Treatment evaluation

**Fig. 2:**
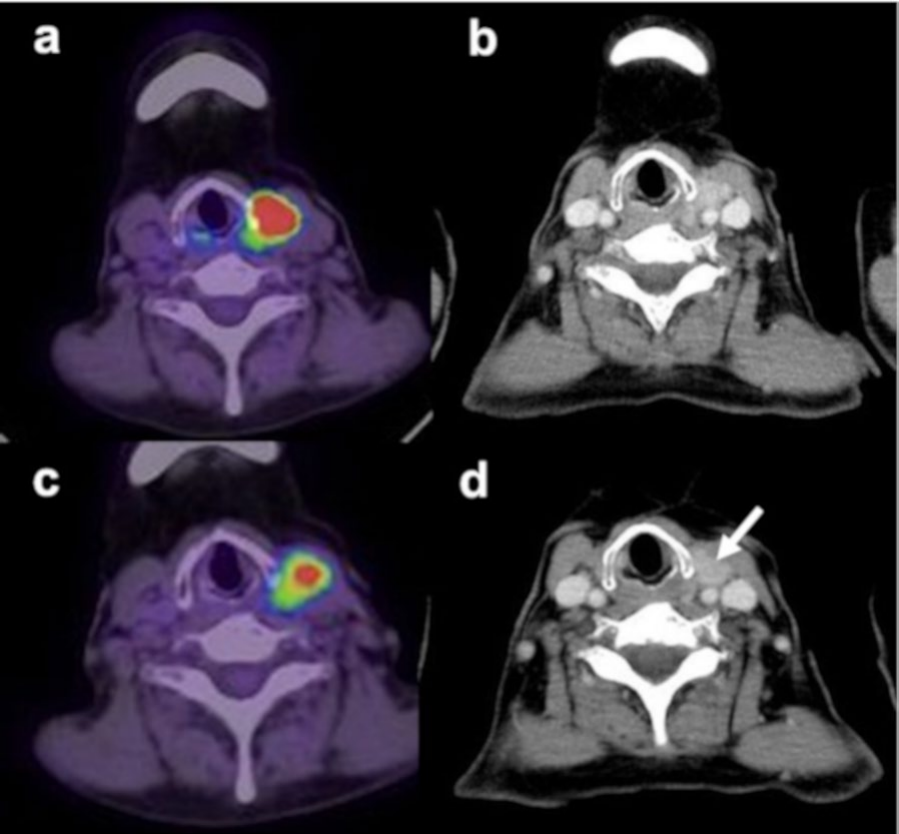
Imaging findings in 67-year-old patient with papillary carcinoma undergoing PET/CT and contrast-enhanced CT. At the initial treatment, PET/CT (**a**) and contrast-enhanced (**b**) showed a 2 cm primary tumour in the left thyroid with an SUVmax of 48.7. Systemic chemotherapy with a lenvatinib dose of 24 mg was initiated as the first-line therapy. After 1 week of treatment with lenvatinib, PET/CT (**c**) demonstrated a remarkable decline in SUVmax to 24.2. However, the mass is still visualised on contrast-enhanced CT (**d**) performed 4 weeks after treatment (arrow). At the time of writing this report, the patient was alive for more than 6 years after treatment. SUVmax: maximum standardised uptake value

**Fig. 3:**
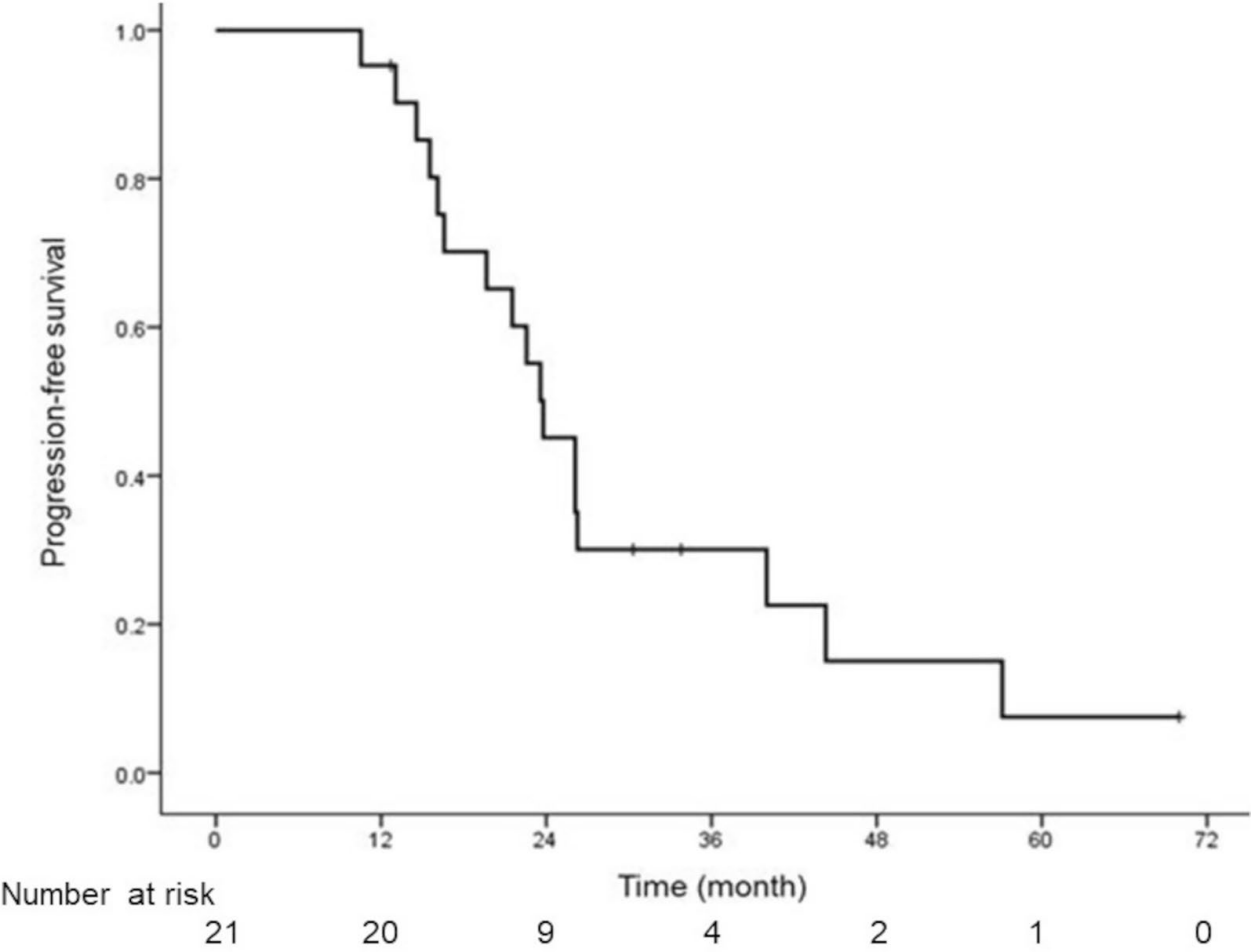
Kaplan–Meier curve for PFS in all patients (n = 21)

As mentioned above, treatment was discontinued in one patient. Lenvatinib treatment (24 mg) was initiated in twenty-one patients. During the first week, the lenvatinib dose was reduced or stopped in three patients because of common terminology criteria for adverse events (CTCAE) grade 3 hypertension. The relative dose intensity during the first week of treatment was 0.95 mg/body/week. After 1 week of treatment, the patients underwent FDG PET/CT. The typical images are shown in Fig. 2. In this patient, FDG PET/CT showed a rapid treatment response after 1 week of treatment, whereas CT performed after 4 weeks of lenvatinib treatment did not show tumour shrinkage. Contrast-enhanced CT was performed at least 4 weeks after treatment initiation, and additional CT for confirmation was performed at least 8 weeks later. Of the 19 patients with target lesions, the response rate was 63% (including one complete response (CR) and 11 partial responses (PR)). Stable disease and progressive disease (PD) were seen in six patients and one patient, respectively. The PFS of all the patients is shown in Fig. 3. The median PFS in all the patients was 23.8 months (95% CI 19.9–27.6 months). The median PFS durations in CR + PR and stable disease + PD were 26.1 months and 16.6 months, respectively (*P* = 0.166).

### ROC curve analysis

**Fig. 4:**
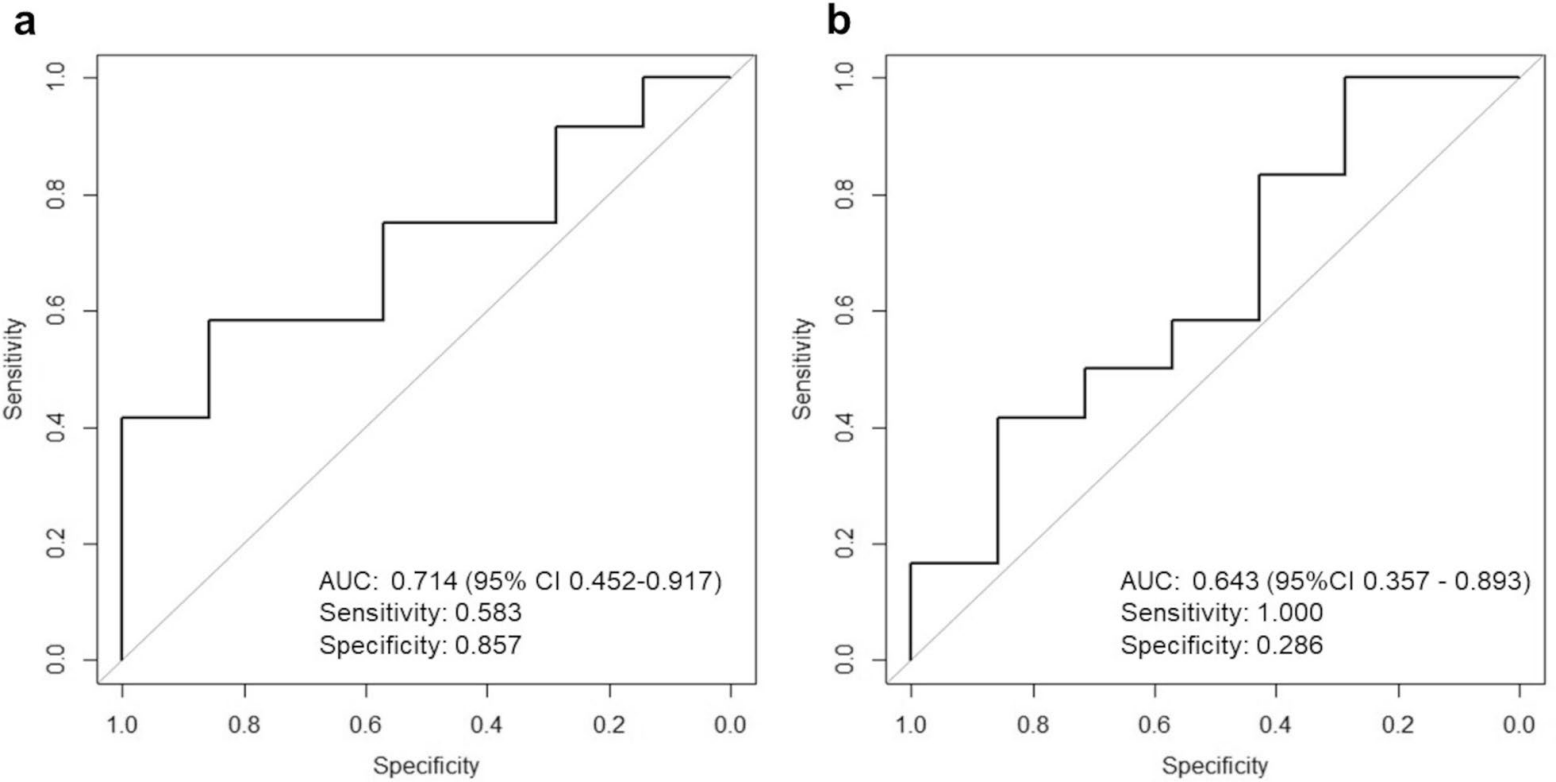
ROC curve for SUVmax (**a**) and TLG (**b**). The AUC for the SUVmax and TLG were 0.714 and 0.643, respectively. FDG PET/CT was performed after 1 week of lenvatinib treatment

ROC curve analysis of SUVmax after 1 week of lenvatinib treatment was performed as the primary endpoint. The ROC curve for SUVmax is shown in Fig. 4a. The AUC was 0.714 (95% CI 0.452–0.917) based on 2000 bootstrap replicates, and the best cut-off value for treatment response (CR + PR) for SUVmax was 15.211. The sensitivity and specificity of this cut-off value were 0.583 and 0.857, respectively.

ROC curve analysis of the TLG was performed as the secondary endpoint (Fig. 4b); AUC was 0.643 (95% CI 0.357–0.893). The best cut-off value for the treatment response for TLG was 772.72. The sensitivity and specificity of this cut-off value were 1.000 and 0.286, respectively.

### PFS analysis

**Fig. 5:**
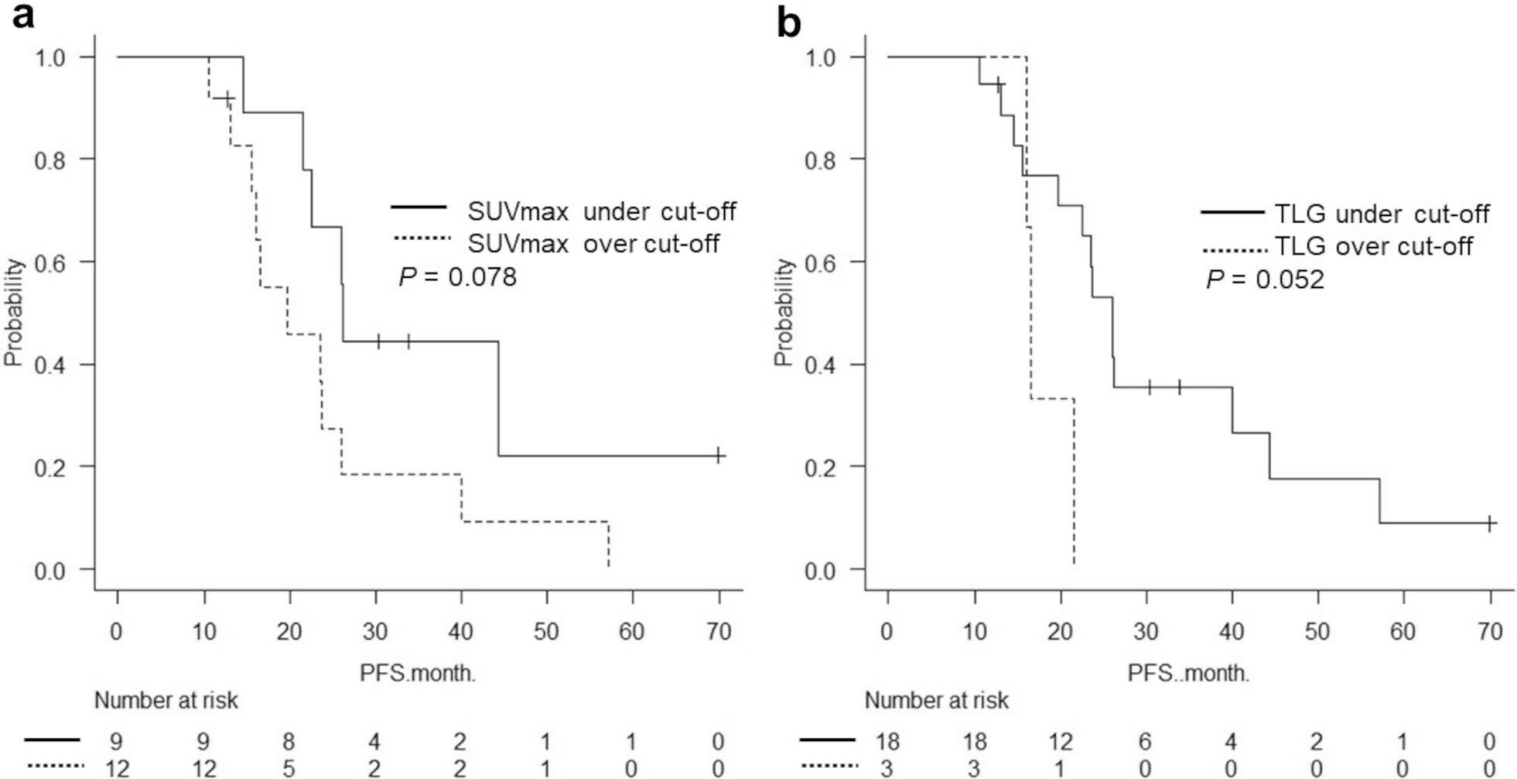
Kaplan–Meier curve for PFS by cut-off value. SUVmax (**a**) and TLG (**b**)

PFS was evaluated based on the results of ROC curve analysis. Patients with an SUVmax cut-off value (15.211) tended to have longer PFS. The median PFS was 26.3 months in patients with an under-cut-off value and 19.7 months in patients with an over-cut-off value (*P* = 0.078, Fig. 5a). Regarding the TLG, only three patients had a TLG cut-off value (772.72). The median PFS was 26.1 months in patients with an under-cut-off value and 16.5 months in those with an over-cut-off value (*P* = 0.052, Fig. 5b).

## Discussion

We attempted to confirm that FDG PET/CT can predict treatment outcome 1 week after the initiation of lenvatinib treatment and to our knowledge, this is the first prospective study in patients with unresectable or advanced DTC refractory to radioiodine treatment. In a Phase III study, PFS with lenvatinib was reported to be 18.3 months [10]. The PFS in DTC is relatively longer than that in other types of cancer; thus, the cost of lenvatinib treatment is elevated, because it costs approximately $15,000 per month. Additionally, lenvatinib has unique adverse effects, such as hypertension, hand-foot syndrome, proteinuria, and thromboembolism, which can lead to both discontinuation of treatment and impairment in quality of life. This study provides insight into the early evaluation of treatment outcomes using FDG PET/CT. If FDG PET/CT can predict treatment outcomes at an early time point after treatment initiation, it can be useful to avoid adverse effects and high treatment costs in advance.

In this study, we focussed on DTC in patients with thyroid cancer. As DTC usually progresses slowly, its duration tends to increase. These patients may benefit from the results of this study, as we aimed to predict treatment outcomes earlier than with conventional CT using FDG PET/CT. Patients with medullary or anaplastic carcinoma were excluded from the study. Medullary carcinomas account for only 3% of thyroid carcinoma [17]. Occasionally, patients with medullary carcinoma are of the familial type or part of the multiple endocrine neoplasia type IIA or IIB. Anaplastic thyroid carcinoma is one of the most aggressive malignancies with poor prognosis. Although rare, representing only 2% of clinically recognised thyroid cancers, the overall median survival is limited to months [18].

At the time of study planning, two oral tyrosine kinase inhibitors, sorafenib and lenvatinib, were available for patients with unresectable and advanced DTC refractory to radioiodine treatment. RR and PFS were reported to be 12.2% and 10.8 months for sorafenib, and 64.8% and 18.3 months for lenvatinib, respectively [9, 10]. There appears to be a relatively large difference in efficacy between these two drugs, although no head-to-head clinical trials have compared sorafenib and lenvatinib. Hence, we chose lenvatinib as the therapeutic drug for this study. In 2020, selpercatinib, a small-molecule rearranged during transfection (RET) kinase inhibitor, demonstrated good efficacy in patients with activating mutations in *RET* or fusions involving the *RET* gene in the LIBRETTO-001 trial [19]. In the clinical trial, 143 of 169 patients had medullary carcinoma, whereas only 13 had papillary carcinoma. Chromosomal rearrangements involving the *RET* proto-oncogene are estimated to be driver mutations in approximately 5–15% of papillary thyroid cancer [20]. Novel drugs have recently been developed for this purpose. Larotrectinib and entrectinib are applicable to patients with neurotrophic receptor tyrosine kinase (*NTRK)* gene fusion-positive advanced solid tumours [21, 22]. Although these drugs have a high response rate, the overall prevalence of *NTRK* fusion-positive tumours is low, at 0.30% among 45 types of cancers [23]. The *BRAF* V600E mutation is the most common mutation, occurring in approximately 45% of patients with papillary carcinoma [24]. Dabrafenib and trametinib combination therapy may be an option for patients positive for the *BRAF* V600E mutation. As of 2022, dabrafenib plus trametinib has not yet been approved for the treatment of thyroid cancer in Japan; therefore, its effects did not impact this study.

The primary endpoint of this study was to evaluate the predictive value of SUVmax obtained using FDG PET/CT performed 1 week after treatment initiation. As FDG PET/CT can detect viable tumour cells by reflecting glucose metabolism, we hypothesised that FDG PET/CT may be a useful tool for evaluating the therapeutic effect earlier than morphological measurements based on contrast-enhanced CT. In this study, we used two PET parameters, SUVmax and TLG. TLG is considered to be a more reflective index of glucose metabolism activity than SUVmax. It compensates for the limitations of SUVmax and reflects the overall metabolic activity of the tumour. Recently, its use has been reported in assessing treatment response and predicting prognosis in patients with head and neck cancer [25]. The obtained AUC from the ROC curve analysis was 0.714 for SUVmax, while TLG had a lower value of 0.643. This lower value for TLG is likely attributed to the limited number of patients. Although several trials have been conducted for early FDG PET/CT evaluation of response in lung and breast cancers [26, 27], a rationale for the timing of FDG PET/CT is lacking. In a Phase I study of lenvatinib, changes in pharmacodynamic biomarkers, including increased vascular endothelial growth factor (VEGF), stromal cell-derived factor-1 alpha (SDF 1α), and decreased soluble VEGF receptor 2 (sVEGFR2) significantly correlated with increasing lenvatinib exposure. These changes were observed 1 week after treatment initiation [28]. Additionally, all FDG PET/CT images were acquired at the Hokkaido University Hospital using the same scanner to eliminate discrepancies between institutions.

Starting with a low dose of lenvatinib can avoid toxicities, such as asthenia, hypertension, weight loss, diarrhoea, nausea, and appetite loss. Brouse et al. showed that a starting lenvatinib dose of 18 mg/day did not demonstrate non-inferiority compared to a starting dose of 24 mg/day, as assessed by the objective response rate at week 24 [29]. All the patients were treated with a starting dose of lenvatinib of 24 mg/day. The relative dose intensity was as high as 0.95 during the first week of treatment. Of the 19 patients with target lesions, 13 (68%) responded to the treatment. Given that the response rate to lenvatinib was 64.8% in a phase 3 study [10], the dose intensity after the first week of treatment was comparable to that of other studies.

Our study had several limitations. First, patient recruitment took longer than was expected. There are two reasons for this finding: DTC is a rare cancer, and FDG PET/CT must be performed using the same device at our institute, despite this being a multicentre study. Second, FDG PET/CT and contrast-enhanced CT were not always performed simultaneously, considering the radiation exposure dose and Japanese national health insurance system. Third, we could not demonstrate a significant difference in DFS using the cut-off value because of the small number of patients, although there was a tendency. Fourth, it is challenging to discontinue lenvatinib treatment for these patients, even if an early treatment response is suggested based on the FDG PET/CT findings obtained 1 week after lenvatinib therapy. This difficulty arises from the limited alternative treatment options available for these patients in their current situation.

## Conclusions

In conclusion, the therapeutic effects of lenvatinib can be detected earlier than those of CT as a decrease in FDG uptake. FDG PET/CT examination 1 week after the initiation of lenvatinib treatment may predict treatment outcomes in patients with DTC. This result warrants further study to prevent adverse effects and high treatment costs.

## Data Availability

Information regarding this study can be found in the UMIN Clinical Trial Registry. Web link: https://www.umin.ac.jp/icdr/index-j.html. The datasets obtained in this study are available from the corresponding author upon reasonable request.

## Abbreviations

DTC: Differentiated thyroid carcinoma
FDG: ^18^F-fluorodeoxyglucose
PET/CT: Positron emission tomography/computed tomography
TSH: Thyroid stimulating hormone
ECOG PS: Eastern Cooperative Oncology Group performance status
RR: Response rate
PFS: Progression-free survival
UMIN: University Hospital Medical Information Network
SUVmax: Maximum standardised uptake value
ROC-AUC: Area under the receiver operating characteristic
TLG: Total lesion glycolysis
MTV: Metabolic tumour volume
RECIST: Response evaluation criteria in solid tumours
VEGF: Vascular endothelial growth factor
SDF 1α: Stromal cell-derived factor-1 alpha
sVEGFR2: Soluble vascular endothelial growth factor receptor 2
RET: Rearranged during transfection
NTRK: Neurotrophic receptor tyrosine kinase
TSH: Thyroid stimulating hormone
FT3: Free triiodothyronine
FT4: Free thyroxine

## References

[1] La Vecchia C, Malvezzi M, Bosetti C, Garavello W, Bertuccio P, Levi F (2015). Thyroid cancer mortality and incidence: a global overview. Int J Cancer.

[2] Ferlay J, Steliarova-Foucher E, Lortet-Tieulent J, Rosso S, Coebergh JW, Comber H (2013). Cancer incidence and mortality patterns in Europe: estimates for 40 countries in 2012. Eur J Cancer..

[3] https://www.nccn.org/store/login/login.aspx?ReturnURL=http://www. NCCN clinical practice guidelines in oncology. Thyroid cancer. http://nccn.org/professionals/physician_gls/pdf/thyroid.pdf. Accessed 15 Feb, 2023.

[4] Lubitz CC, Kong CY, McMahon PM, Daniels GH, Chen Y, Economopoulos KP (2014). Annual financial impact of well-differentiated thyroid cancer care in the United States. Cancer.

[5] Megwalu UC, Moon PK (2022). Thyroid cancer incidence and mortality trends in the United States: 2000–2018. Thyroid.

[6] Shoup M, Stojadinovic A, Nissan A, Ghossein RA, Freedman S, Brennan MF (2003). Prognostic indicators of outcomes in patients with distant metastases from differentiated thyroid carcinoma. J Am Coll Surg.

[7] Kelil T, Keraliya AR, Howard SA, Krajewski KM, Braschi-Amirfarzan M, Hornick JL (2016). Current concepts in the molecular genetics and management of thyroid cancer: an update for radiologists. Radiographics.

[8] Gild ML, Bullock M, Robinson BG, Clifton-Bligh R (2011). Multikinase inhibitors: a new option for the treatment of thyroid cancer. Nat Rev Endocrinol.

[9] Brose MS, Nutting CM, Jarzab B, Elisei R, Siena S, Bastholt L (2014). Sorafenib in radioactive iodine-refractory, locally advanced or metastatic differentiated thyroid cancer: a randomised, double-blind, phase 3 trial. Lancet.

[10] Schlumberger M, Tahara M, Wirth LJ, Robinson B, Brose MS, Elisei R (2015). Lenvatinib versus placebo in radioiodine-refractory thyroid cancer. N Engl J Med.

[11] Stjepanovic N, Capdevila J (2014). Multikinase inhibitors in the treatment of thyroid cancer: specific role of lenvatinib. Biologics.

[12] Hirata K, Kobayashi K, Wong KP, Manabe O, Surmak A, Tamaki N (2014). A semi-automated technique determining the liver standardized uptake value reference for tumor delineation in FDG PET-CT. PLoS ONE.

[13] Wahl RL, Jacene H, Kasamon Y, Lodge MA (2009). From RECIST to PERCIST: evolving considerations for PET response criteria in solid tumors. J Nucl Med.

[14] Shankar LK, Hoffman JM, Bacharach S, Graham MM, Karp J, Lammertsma AA (2006). Consensus recommendations for the use of ^18^F-FDG PET as an indicator of therapeutic response in patients in National Cancer Institute Trials. J Nucl Med.

[15] Obuchowski NA, McClish DK (1997). Sample size determination for diagnostic accuracy studies involving binormal ROC curve indices. Stat Med.

[16] Staub Y, Nishiyama A, Suga Y, Fujita M, Matsushita R, Yano S (2019). Clinical characteristics associated with Lenvatinib-induced Fistula and Tumor-related bleeding in patients with thyroid cancer. Anticancer Res.

[17] Kebebew E, Clark OH (2000). Medullary thyroid cancer. Curr Treat Options Oncol.

[18] Pasieka JL (2003). Anaplastic thyroid cancer. Curr Opin Oncol.

[19] Wirth LJ, Sherman E, Robinson B, Solomon B, Kang H, Lorch J (2020). Efficacy of selpercatinib in RET-altered thyroid cancers. N Engl J Med.

[20] Kato S, Subbiah V, Marchlik E, Elkin SK, Carter JL, Kurzrock R (2017). RET aberrations in diverse cancers: next-generation sequencing of 4,871 patients. Clin Cancer Res.

[21] Hong DS, DuBois SG, Kummar S, Farago AF, Albert CM, Rohrberg KS (2020). Larotrectinib in patients with TRK fusion-positive solid tumours: a pooled analysis of three phase 1/2 clinical trials. Lancet Oncol.

[22] Doebele RC, Drilon A, Paz-Ares L, Siena S, Shaw AT, Farago AF (2020). Entrectinib in patients with advanced or metastatic NTRK fusion-positive solid tumours: integrated analysis of three phase 1–2 trials. Lancet Oncol.

[23] Westphalen CB, Krebs MG, Le Tourneau C, Sokol ES, Maund SL, Wilson TR (2021). Genomic context of NTRK1/2/3 fusion-positive tumours from a large real-world population. NPJ Precis Oncol.

[24] Yarchoan M, LiVolsi VA, Brose MS (2015). BRAF mutation and thyroid cancer recurrence. J Clin Oncol.

[25] Pak K, Cheon GJ, Nam HY, Kim SJ, Kang KW, Chung JK (2014). Prognostic value of metabolic tumor volume and total lesion glycolysis in head and neck cancer: a systematic review and meta-analysis. J Nucl Med.

[26] Lin NU, Guo H, Yap JT, Mayer IA, Falkson CI, Hobday TJ (2015). Phase II study of Lapatinib in combination with trastuzumab in patients with human epidermal growth factor receptor 2-positive metastatic breast cancer: clinical outcomes and predictive value of early [18F]Fluorodeoxyglucose positron emission tomography imaging (TBCRC 003). J Clin Oncol.

[27] Sunaga N, Oriuchi N, Kaira K, Yanagitani N, Tomizawa Y, Hisada T (2008). Usefulness of FDG-PET for early prediction of the response to gefitinib in non-small cell lung cancer. Lung Cancer.

[28] Koyama N, Saito K, Nishioka Y, Yusa W, Yamamoto N, Yamada Y (2014). Pharmacodynamic change in plasma angiogenic proteins: a dose-escalation phase 1 study of the multi-kinase inhibitor lenvatinib. BMC Cancer.

[29] Brose MS, Panaseykin Y, Konda B, de la Fouchardiere C, Hughes BGM, Gianoukakis AG (2022). A randomized study of lenvatinib 18 mg vs 24 mg in patients with radioiodine-refractory differentiated thyroid cancer. J Clin Endocrinol Metab.

